# Ankle Sprains in Brazilian Professional Soccer: Epidemiological Analysis of 126,357 Match Hours *

**DOI:** 10.1055/s-0044-1785660

**Published:** 2024-05-13

**Authors:** Ewerton Borges de Souza Lima, Gabriel de Melo Salgado, Eduardo Patrício Mello, Paulo Henrique Schmidt Lara, Gustavo Gonçalves Arliani, Moisés Cohen

**Affiliations:** 1 Departamento de Ortopedia e Traumatologia, Centro de Traumatologia Esportiva, Escola Paulista de Medicina, Universidade Federal de São Paulo, São Paulo, SP, Brasil; 2 Departamento de Ortopedia e Traumatologia, Escola Paulista de Medicina, Universidade Federal de São Paulo, São Paulo, SP, Brasil

**Keywords:** ankle injuries, epidemiology, soccer, sports, sports medicine

## Abstract

**Objective** This study aimed to perform an epidemiological analysis of ankle sprains in professional soccer players in Brazil.

**Methods** This prospective epidemiological study analyzed ankle sprains in professional male soccer athletes during the Brazilian Championship and the Paulista Football Championship from 2016 to 2019. All on-pitch medical care provided in official matches was recorded. The variables evaluated included the age and position of the player, injury diagnosis, pitch site where the injury occurred, playing time, imaging tests performed, surgical treatment, time away from competitions, and injury recurrence. We assessed the incidence of injuries according to the *Federation Internationale de Football Association* (FIFA) incidence formula.

**Results** Ankle sprains represented 10.17% of total injuries, with a FIFA index equal to 2,002. Lateral ligament injuries represented 53.75% of all sprains. The forwards were the most injured athletes, with 86 lesions. The midfield had the highest number of sprains (75.50%). Almost half (47.04%) of sprains occurred in the final 15 minutes of each half-time. Injuries recurred in 31.22% of cases, and 7.11% of injuries underwent surgical treatment. The average time away from competitions was 13.95 days.

**Conclusions** Ankle sprains are common injuries in soccer. Although the average time to return to sport is brief, these injuries have a high recurrence rate and are potentially surgical, leading to a longer time off competitions.

## Introduction

Football is the most popular sport in the world, with 240 million amateur athletes and around 200,000 professional ones. 1 Added to its popularity, it has a high rate of injuries. In recent years, the physical demands have increased, forcing athletes to work close to their maximum exhaustion limits, increasing the predisposition to injuries.

Several movements occur during a football match, requiring sudden changes in direction, on average, every 6 seconds. 2 In this scenario, the injury mechanisms favor the involvement of the lower limbs, especially the ankles. These lesions correspond to around 13% of injuries in football, only behind thigh and knee injuries. 3 In addition to the impact on the athlete's career, these injuries result in high costs for clubs due to treatment and rehabilitation. Understanding the epidemiology and incidence of ankle injuries can help clubs prepare their athletes in a targeted manner. Therefore, this study aimed to perform an epidemiological analysis of ankle sprains in professional football players in the main Brazilian championships from 2016 to 2019.

## Materials and Methods

This prospective epidemiological study evaluated ankle sprains in professional male football athletes in two divisions of two men's football championships with significant social and economic relevance in Brazil, that is, the Brazilian Football Championship (CBF, for Campeonato Brasileiro de Futebol in Portuguese) and Paulista Football Championship (CPF, for Campeonato Paulista de Futebol), from 2016 to 2019. The Research Ethics Committee approved this study under registration CAAE 56723616.3.0000.5505. All participants included in the study signed the informed consent form.

All on-pitch medical care provided during official matches was recorded. Club doctors received training to fill out two forms on two online platforms, Transfermarkt (Transfermarkt GmbH & Co. KG, Hamburg, Germany) and SurveyMonkey (Momentive AI., San Mateo, CA, USA). The first form was filled out immediately after each match, and the second form was filled out after the athlete returned to the sport. The collection occurred throughout the championship, and we contacted the doctors monthly to remind them to fill out the forms.

All players with injuries resulting from ankle sprains were included in the study, regardless of age. The variables evaluated were age and position of the player, injury diagnosis, laterality, pitch site where the injury occurred, playing time, imaging tests performed, surgical treatment, time away from the competition, and injury recurrence. For this study, injury was defined as a musculoskeletal complaint occurring during a match that resulted in a player missing at least one match or training session. We assessed the incidence of injuries using the FIFA incidence formula = (total injuries x 1,000)/total hours played.

The descriptive statistical analysis of quantitative variables was performed in Excel 2016 (Microsoft Corporation, Redmond, WA, USA). We used the IBM SPSS Statistics for Windows Version 20.0 software (IBM Corp., Armonk, NY, USA) for the statistical inference of continuous variables. Qualitative variables were analyzed using the chi-square test, and the analysis of variance (ANOVA) test was used for multivariate analysis, defining a 95% confidence interval and a 5% significance ( *p* < 0.05). The Shapiro-Wilk test determined the normality of the sample.

The study took place during 4 CBF seasons, with the participation of 20 clubs per year in each division. Each club played 38 matches per season. In the same period, there were 4 CPF seasons, with the participation of 16 to 20 clubs per year in each division. Each club played 17 to 22 matches per season. In total, we analyzed 15 seasons from 2016 to 2019 (there was no record from the CBF second division in 2019), totaling 3,828 matches and 126,357 match hours.

## Results

During the study, there were 7,899 on-pitch care visits, of which 2,486 resulted in the diagnosis of injuries. Of these, 253 ankle sprains were recorded (10.17% of total injuries) with a FIFA index equal to 2,002. Over the years, the total number of injuries progressively dropped from 86 in 2016 to 38 in 2019.

### Diagnoses, Age, and Supplementary Tests

We identified six different diagnoses of ankle sprain injuries during the championships. The most common injuries were lateral ligament lesions (LLLs), which represented 53.75% of total sprains, followed by simple sprains (sprains with no diagnosis of ligament injury, fractures, or other associated injuries), accounting for 28.85% of cases. Other diagnoses include medial ligament lesions (MLLs), unimalleolar fractures, bimalleolar fractures, and syndesmosis injuries ( Table 1 ).

**Table 1:** Main characteristics, pitch site where the injuries occurred, and their relationships with the diagnoses and positions of the players

Diagnosis	n (%)	FIFA index	Recurrence	Surgery	Time away	Pitch site			
						Attack area	Attack midfield	Defense midfield	Defensive area
Simple sprain	73 (28.85%)	0.578	28 (11.07%)	0	6.78	19 (7.51%)	21 (8.30%)	22 (8.70%)	45 (17.79%)
Unimalleolar ankle fracture	8 (3.16%)	0.016	0	6 (2.37%)	105	3 (1.19%)	6 (2.37%)	3 (1.19%)	9 (3.56%)
Bimalleolar ankle fracture	2 (0.79%)	0.063	0	2 (0.79%)	89	0	2 (0.79%)	0	0
Lateral ligament lesion	136 (53.75%)	1.076	44 (17.39%)	5 (1.98%)	10.44	1 (0.40%)	0	0	1 (0.40%)
Medial ligament lesion	26 (10.28%)	0.206	6 (2.37%)	2 (0.79%)	16.27	0	0	1 (0.40%)	3 (1.19%)
Syndesmosis injury	8 (3.16%)	0.063	1 (0.40%)	3 (1.19%)	37.75	8 (3.16%)	7 (2.77%)	8 (3.16%)	15 (5.93%)
**Position**									
Striker	86 (33.99%)	0.680	23 (9.09%)	5 (1.98%)	10.92	23 (9.09%)	32 (12.65%)	30 (11.86%)	3 (1.19%)
Midfielder	44 (17.39%)	0.348	17 (6.72%)	4 (1.58%)	15.86	1 (0.40%)	16 (6.32%)	23 (9.09%)	4 (1.58%)
Defensive midfielder	33 (13.04%)	0.261	8 (3.16%)	2 (0.79%)	13.52	0	7 (2.77%)	25 (9.88%)	1 (0.40%)
Wing	50 (19.76%)	0.396	17 (6.72%)	4 (1.58%)	14.32	4 (1.58%)	4 (1.58%)	25 (9.88%)	17 (6.72%)
Defender	30 (11.85%)	0.237	9 (3.56%)	3 (1.19%)	22.50	1 (0.40%)	10 (3.95%)	15 (5.93%)	4 (1.58%)
Goalkeeper	10 (3.95%)	0.079	5 (1.98%)	0 (0%)	5.6	0	1 (0.40%)	3 (1.19%)	6 (2.37%)

Note: Percentages referred to the total number of ankle injuries assessed. The average age is given in years. The average time away from competition is given in days.

The average age of the injured players was 25.94 years old. Different injuries had a similar distribution between age groups. Imaging exams were requested in 94.07% of the lesions, totaling 391 exams performed ( Table 2 ).

**Table 2:** Imaging tests requested for each injury diagnosis

Diagnosis	None	MRI	X-ray	X-ray + MRI	X-ray + MRI + CT	Total
Simple sprain	15	9	48	1	0	73
Unimalleolar ankle fracture	0	0	2	0	0	2
Bimalleolar ankle fracture	0	0	8	0	0	8
Lateral ligament lesion	0	8	0	127	1	136
Medial ligament lesion	0	4	0	22	0	26
Syndesmosis injury	0	3	4	1	0	8
Total	15	24	62	151	1	253

Abbreviations: MRI, magnetic resonance imaging; X-ray, radiography; CT, computed tomography.

### Player Position, Pitch Site, and Time of Injury

The position with the highest number of injuries was striker, followed by wings. The positions with the lowest number of ankle injuries were goalkeepers and defenders ( Table 1 ). Regarding the total number of injured players from the same position, ankle sprains were more prevalent in midfielders ( Table 3 ).

**Table 3:** Position of players with ankle injuries

Diagnosis	Position					
	Striker	Midfielder	Defensive midfielder	Wing	Defender	Goalkeeper
Simple sprain	25 (9.88%)	12 (4.74%)	6 (2.37%)	16 (6.32%)	8 (3.16%)	6 (2.37%)
Bimalleolar ankle fracture	0	1 (0.40%)	6 (5.61%)	0	1 (0.40%)	0
Unimalleolar ankle fracture	2 (0.79%)	1 (0.40%)	0	3 (1.19%)	1 (0.40%)	0
Lateral ligament lesion	46 (18.18%)	21 (8.30%)	0	26 (10.28%)	16 (6.32%)	3 (1.19%)
Medial ligament lesion	11 (4.35%)	3 (1.19%)	0	3 (1.19%)	4 (1.58%)	1 (0.40%)
Syndesmosis injury	2 (0.79%)	1 (0.40%)	5 (1.98%)	2 (0.79%)	0	0

The midfield had the highest number of sprains (75.50%). Only 13.04% of injuries occurred in the defense area, and 11.46% in the attack area ( Table 1 ). The player's position was statistically associated with the place on the pitch where the injury occurred ( *p* < 0.001), with the defensive midfield being the most common place for sprains for all positions, except for strikers (who presented more sprains in the attack area) and goalkeepers (who presented more sprains in the defensive area). The end of each half-time had the highest number of injuries, and 47.04% of lesions occurred between 31 and 45 minutes and 75 and 90 minutes ( Table 4 ).

**Table 4:** Relationship between match time at injury occurrence and player position. Min, Minutes.

Position	Match time							
	0–15 min	16–30 min	31–45 min	1 ^st^ extra time	46–60 min	61–75 min	76–90 min	2 ^nd^ extra time
Striker	4 (1.58%)	14 (5.53%)	20 (7.91%)	10 (3.95%)	4 (1.58%)	10 (3.95%)	22 (8.70%)	2 (0.79%)
Midfielder	6 (2.37%)	8 (3.16%)	9 (3.56%)	1 (0.40%)	3 (1.19%)	6 (2.37%)	9 (3.56%)	0
Defensive midfielder	2 (0.79%)	5 (1.98%)	6 (2.37%)	0	6 (2.37%)	1 (0.40%)	12 (4.74%)	0
Wing	5 (1.98%)	6 (2.37%)	16 (6.32%)	5 (1.98%)	4 (1.58%)	4 (1.58%)	9 (3.56%)	1 (0.40%)
Defended	4 (1.58%)	2 (0.79%)	5 (1.98%)	3 (1.19%)	3 (1.19%)	8 (3.16%)	5 (1.98%)	0
Goalkeeper	1 (0.40%)	1 (0.40%)	1 (0.40%)	1 (0.40%)	1 (0.40%)	0	5 (1.98%)	0
Total	22 (8.70%)	36 (14.23%)	57 (22.53%)	20 (7.91%)	21 (8.30%)	25 (9.88%	62 (24.51%)	3 (1.19%

### Recurrence, Surgery, and Time Away from Competitions

Injuries recurred in 31.22% of cases. Simple sprains (38.36%) and LLLs (32.35%) had the highest recurrence rates, while fractures did not recur ( Table 1 ).

Injuries underwent surgical treatment in 7.11% of cases. Unimalleolar ankle fractures represented 33.33% of surgeries. No simple ankle sprain received surgical treatment, and only 3.68% of LLLs and 7.69% of LLMs required surgery. Recurrent injuries were treated surgically in 12.65% of cases, in contrast to 80% of non-recurrent injuries ( Table 1 ).

Injuries were classified per severity according to the athlete's absence time, as follows: mild (< 3 days), minor (3–7 days), moderate (8–28 days), major (4–8 weeks), and severe (> 8 weeks) injuries. The average time off competition was 13.95 days, with severe and major injuries accounting for 4.34% and 3.56% of total injuries, respectively ( Table 5 ).

**Table 5:** Severity of ankle injuries

Diagnosis	Severity				
	Mild	Minor	Moderate	Major	Severe
Simple sprain	38 (15.02%)	15 (5.93%)	20 (7.91%)	0	0
Bimalleolar ankle fracture	0	0	0	0	2 (0.79%)
Unimalleolar ankle fracture	0	1 (0.40%)	0	1 (0.40%)	6 (2.37%)
Lateral ligament lesion	32 (12.65%)	44 (17.39%)	55 (21.74%)	4 (1.58)	1 (0.40%)
Medial ligament lesion	4 (1.58%)	10 (3.95%)	0	1 (0.40%)	1 (0.40%)
Syndesmosis injury	0	0	3 (1.19%)	4 (1.58%)	1 (0.40%)
Total	74 (29.25%)	70 (27.67%)	78 (30.83%)	10 (3.95%)	11 (4.35%)

## Discussion

The main findings of the present study were the high incidence of ankle sprains over 4 years. Furthermore, sprains mainly affected strikers and midfielders, with higher occurrence in midfield and the final 15 minutes of each half-time. The recurrence rate was high (31.22%), and the average time away from competitions was around 2 weeks.

Ekstrand et al. 4 stated that ankle sprains accounted for 17 to 21% of all sports injuries, showing their great representation. Our study identified a FIFA index of 2.00 for ankle sprains, a value higher than upper limb injuries (FIFA index = 1.34) 5 and ruptures of the anterior cruciate ligament (FIFA index = 0.41), 6 but lower than muscle injuries (FIFA index = 7.66). 7

A German study with Bundesliga (the German professional football league) athletes observed 280 episodes of ankle sprains (5.17% of total injuries) from the 2008 to 2014 seasons. This study reported a rate of 46.66 injuries per year, which represents a difference of 35.55% concerning our study. 3 Árnason et al. 8 evaluated 10 elite Icelandic football clubs, and 82% of the injuries occurred in the lower limbs.

Dauty et al. 9 found no difference in the incidence of injuries during the 15 seasons analyzed between 1995 and 2010, contrasting with our results. There is no reported reason for the lower number of injuries in our study. However, over the years, the quality of prematch physical preparation, physical therapy, pitch quality, and injury prevention treatments significantly improved in Brazilian football, potentially contributing to a reduction in injuries. 2 10

Evaluating the distribution of injuries by month, Kofotolis et al. 11 observed that 47.48% of ankle sprains happened in August and September. Morgan and Oberlander 12 observed that 24% of lesions occurred in the first third of the season, 25% in the second third, and 29% in the last third of the season, which also conflicts with our data.

For Nery et al., 13 medial and lateral ligament injuries represented 66.8% of the total number of ankle injuries during the 2 seasons evaluated. Compared with our study, 63.93% of all ankle injuries were medial and lateral complex lesions, consistent with the statement that medial and lateral complex injuries are common in ankle sprains.

Árnason et al. 8 noted that 29% of injuries resulted from muscle lesions, 22% from ligament lesions, and 20% from direct blunt trauma. Most ankle injuries presented lateral ankle complex lesions.

In a systematic review of all ankle injuries in football, 76.8% were ankle sprains, which is the most common cause of ankle injuries, consistent with our study. 4 Nery et al. 13 indicated that the most common mechanism of trauma in professional football athletes was direct player-to-player trauma (32%), with excessive use (intense joint mobility at the extreme of the range of motion) associated with 26% of the causes of ankle injuries. Kofotolis et al. 11 also stated that most ankle injuries occurred through direct contact with another athlete, with a 63.3% rate, while 36.7% of lesions did not result from contact. At the 2014 World Cup, 64.4% of athletes' injuries were due to contact. 14

Striker was the athlete's position on the pitch with most ankle sprains. However, considering midfielders and defensive midfielders together, the rate of simple sprains is higher (7.11%). Cohen et al. 2 compared athletes' total body injuries and observed that the midfield position had the highest number of injuries Leventer et al. 15 and Morgan and Oberlander 12 showed that midfielders had 37.6% of total body injuries in the Major League of Soccer (MLS).

Dauty et al. 9 found no difference in the number of any injury type regarding pitch positions; however, there was a slight tendency towards defenders. Kofotolis et al. observed more injuries in defenders and wings (42.4%) compared to midfielders (32.3%) and strikers (20.8%). 11

Kofotolis et al. 11 analyzed that 61.1% of ankle injuries occurred in the final 15 minutes of both half-times, agreeing with our data. Hawkins et al. 16 observed this same pattern.

In the present study, 31.22% of injuries were recurrent. Árnason et al. 8 observed that 35% of injuries were recurrent, with 8% occurring less than 1 month after the previous lesion. Kofotolis et al. found that 60.5% of athletes had recurrent injuries. 11 Football players with a history of previous ankle sprains are 4.9 times more likely to have a recurrent lesion. 13

Cohen et al. 2 evaluated episodes of sprain, with 5.2% (n = 9) of the cases progressing to some surgical procedure from a total of 173 cases. According to Nery et al., 13 20% of athletes with acute ankle sprains will develop mechanical or functional ankle instability, resulting in chronic ankle instability and requiring surgical treatment.

Kofotolis et al. 11 also evaluated the time away from competitions. These authors noted that simple sprains represented the most common injury with less than seven missed sessions. Injury to the anterior tibiotalar ligament resulted in the highest absence rate and 58.7% of total time lost due to lesion.

Morgan and Oberlander 12 classified the severity of the injury into three categories. Of the 256 injuries found, 60% were mild, 26% were moderate, and 14% were severe. Árnason et al. 8 classified injuries into 4 categories according to the time away from competition, and most athletes returned to the sport in less than 2 weeks. Cohen et al. 2 observed that sprains resulted in absence for less than 7 days in 49.7% of cases, from 7 to 30 days in 45%, and over 30 days in 5.2% of cases. All these latter injuries underwent surgical treatment.

The main limitation of this study is the potential data collection bias inherent to epidemiological studies since club doctors were responsible for filling in the data on the platform. During this period, there were some changes of doctors in clubs. To reduce data collection bias, all doctors received training to complete the survey. We were aware of all club doctors' replacements and trained the new doctors. Another limitation is the lack of consensus on the definition of injury in the literature, hindering the comparison of our results with those of other studies. Furthermore, we did not collect data from injuries occurring during training sessions. Lastly, the failure to evaluate the pitch quality and their relationship with sprains was another limitation of the study. Despite these limitations, this is an extensive prospective study lasting 4 years and with a large sample of athletes and injuries which collected unprecedented data from two major professional football championships in Brazil.

## Conclusion

Ankle sprains are common injuries in football. Although the average time to return to sport is brief, these injuries have a high recurrence rate and are potentially surgical, leading to a longer time away from competitions. The most common injuries were LLLs and simple sprains. The most affected positions were striker and midfielder, with higher occurrence in the midfield and the final 15 minutes of each half-time.
