# Supplementary material for: Ankle Sprains in Brazilian Professional Soccer: Epidemiological Analysis of 126,357 Match Hours
Source: Rev Bras Ortop (Sao Paulo). 2024 May 13;59(5):e682–8. doi: 10.1055/s-0044-1785660 (PMC11624919; doi:10.1055/s-0044-1785660)
Supplement: Related texts

# Research article: Entorses do tornozelo no futebol profissional brasileiro: Análise epidemiológica de 126.357 horas de jogo

**Keywords:** epidemiologia, esportes, futebol, medicina do esporte, traumatismos do tornozelo

## Abstract

**Objetivo** Realizar análise epidemiológica das entorses de tornozelo em jogadores profissionais de futebol no Brasil.

**Métodos** Estudo epidemiológico prospectivo das entorses de tornozelo em atletas profissionais de futebol masculino ocorridas no Campeonato Brasileiro e no Campeonato Paulista de Futebol, entre 2016 e 2019. Foram registrados todos os atendimentos médicos em campo realizados durante partidas oficiais. As variáveis avaliadas foram: idade e posição do jogador, diagnóstico da lesão, lateralidade, local em campo onde ocorreu a lesão, tempo de jogo, exames de imagem realizados, realização de tratamento cirúrgico, tempo de afastamento e recorrência da lesão. A incidência das lesões foi avaliada de acordo com a fórmula de incidência da *Federation Internationale de Football Association* (FIFA).

**Resultados** As entorses de tornozelo representaram 10,17% do total de lesões, com índice FIFA = 2.002. As lesões ligamentares laterais representaram 53,75% do total das entorses. Os atacantes foram os mais lesionados, com 86 lesões. O meio de campo foi o local com maior número de entorses (75,50%). Nos 15 minutos finais de cada tempo, ocorreram 47,04% das entorses. As lesões foram reincidentes em 31,22% dos casos, e 7,11% das lesões foram tratadas cirurgicamente. O tempo médio de afastamento foi de 13,95 dias.

**Conclusões** As entorses de tornozelo são lesões frequentes no futebol. Apesar do tempo médio para retorno ao esporte ser breve, essas lesões apresentam grande taxa de reincidência e são potencialmente cirúrgicas, o que está associado a maior tempo de afastamento.

## Introdução

O futebol é o esporte mais popular do mundo, com 240 milhões de atletas amadores e cerca de 200.000 profissionais. 1 Somada à sua popularidade, apresenta alto índice de lesões. Nos últimos anos, a exigência física tem aumentado, o que obriga os atletas a trabalharem perto de seus limites máximos de exaustão, com maior predisposição às lesões.

Os movimentos realizados no futebol são muito variados, exigindo mudanças repentinas de direção, em média, a cada seis segundos. 2 Nesse cenário, os mecanismos de lesão são favoráveis ao acometimento dos membros inferiores, especialmente dos tornozelos. Essas correspondem a cerca de 13% das lesões no futebol, atrás apenas de lesões na coxa e no joelho. 3 Além do impacto na carreira do atleta, essas lesões resultam em altos custos para os clubes, devido ao tratamento e reabilitação. O entendimento da epidemiologia e incidência das lesões do tornozelo pode ajudar as equipes a prepararem seus atletas de forma direcionada. Dessa forma, o objeto deste estudo é realizar uma análise epidemiológica das entorses de tornozelo em jogadores profissionais de futebol, nos principais campeonatos nacionais, ocorridos entre 2016 e 2019.

## Materiais e Métodos

Trata-se de um estudo epidemiológico prospectivo que avaliou as entorses de tornozelo em atletas profissionais de futebol masculino em duas divisões de dois campeonatos de futebol masculino com importante relevância social e econômica no Brasil - Campeonato Brasileiro de Futebol (CBF) e Campeonato Paulista de Futebol (CPF) - durante os anos de 2016 a 2019. O estudo foi aprovado pelo Comitê de Ética em Pesquisa sob o registro CAAE 56723616.3.0000.5505 e o consentimento informado foi obtido de todos os participantes individuais incluídos no estudo.

Foram registrados todos os atendimentos médicos em campo realizados durante partidas oficiais. Os médicos dos clubes foram treinados a preencher dois formulários em duas plataformas online, Transfermarkt (Transfermarkt GmbH & Co. KG, Hamburgo, Alemanha) e SurveyMonkey (Momentive AI., San Mateo, CA, EUA). O primeiro formulário era preenchido logo após cada partida e o segundo após o retorno do atleta ao esporte. A coleta foi realizada durante todo o campeonato e mensalmente era feito contato com os médicos, lembrando-os do preenchimento dos formulários.

Todos os jogadores com lesões decorrentes de entorses do tornozelo foram incluídos no estudo, independentemente da idade. As variáveis avaliadas foram: idade e posição do jogador, diagnóstico da lesão, lateralidade, local em campo em que ocorreu a lesão, tempo de jogo, exames de imagem realizados, realização de tratamento cirúrgico, tempo de afastamento e recorrência da lesão. Para este estudo, a lesão foi definida como uma queixa musculoesquelética ocorrida durante um jogo que levou o jogador ao afastamento de pelo menos um jogo ou sessão de treino. A incidência das lesões foi avaliada de acordo com a fórmula de incidência da FIFA = (total de lesões x 1.000)/total de horas de jogo.

O software utilizado para realizar a análise estatística descritiva das variáveis quantitativas foi o Excel 2016 (Microsoft Corporation, Redmond, WA, EUA). O software IBM SPSS *Statistics for Windows* , Versão 20.0 (IBM Corp., Armonk, NY, EUA) foi utilizado para a realização de inferência estatística de variáveis contínuas. Variáveis qualitativas foram analisadas utilizando o teste de qui-quadrado, e o teste de análise de variância (ANOVA) foi utilizado para análise multivariada, definindo o intervalo de confiança de 95% e significância de 5% ( *p* < 0,05). O teste de Shapiro-Wilk foi utilizado para definição da normalidade da amostra.

O estudo aconteceu durante 4 temporadas do CBF, com a participação de 20 clubes por ano em cada divisão, que jogaram 38 partidas por temporada. No mesmo período ocorreram 4 temporadas do CPF, com participação de 16 a 20 clubes por ano em cada divisão, que jogaram 17 a 22 partidas por temporada. No total, foram analisadas 15 temporadas entre 2016 e 2019 (não houve registro da segunda divisão do CBF em 2019), totalizando 3.828 partidas e 126.357 horas de jogo.

## Resultados

Durante o estudo foram registrados 7.899 atendimentos em campo, dos quais 2.486 resultaram em diagnóstico de lesões. Desses, foram registradas 253 entorses de tornozelo (10,17% do total de lesões) com índice FIFA = 2.002. Ao longo dos anos, houve uma redução progressiva no total de lesões, de 86 em 2016 para 38 em 2019.

## Diagnósticos, Idade e Exames Complementares

Seis diferentes diagnósticos de lesões por entorse de tornozelo foram identificados no decorrer dos campeonatos, com destaque para as lesões ligamentares laterais (LLLs), que representaram 53,75% do total dos entorse, seguidas pelas entorses simples (entorse sem diagnóstico de lesão ligamentar, fraturas ou outras lesões associadas), que representaram 28,85% dos casos. Outros diagnósticos foram: lesões ligamentares mediais (LLMs), fraturas unimaleolares, fraturas bimaleolares e lesões da sindesmose ( Tabela 1 ).

**Tabela 1:** Principais características, local no campo em que ocorreram as lesões e suas relações com os diagnósticos e posições dos jogadores

Diagnóstico	n (%)	Incidência FIFA	Reincidência	Cirurgia	Afastamento	Local em campo			
						Área ataque	Meio ataque	Meio defesa	Área defesa
Entorse simples	73 (28,85%)	0,578	28 (11,07%)	0	6,78	19 (7,51%)	21 (8,30%)	22 (8,70%)	45 (17,79%)
Fratura do tornozelo unimaleolar	8 (3,16%)	0,016	0	6 (2,37%)	105	3 (1,19%)	6 (2,37%)	3 (1,19%)	9 (3,56%)
Fratura do tornozelo bimaleolar	2 (0,79%)	0,063	0	2 (0,79%)	89	0	2 (0,79%)	0	0
Lesão ligamentar lateral	136 (53,75%)	1,076	44 (17,39%)	5 (1,98%)	10,44	1 (0,40%)	0	0	1 (0,40%)
Lesão ligamentar medial	26 (10,28%)	0,206	6 (2,37%)	2 (0,79%)	16,27	0	0	1 (0,40%)	3 (1,19%)
Lesão da sindesmose	8 (3,16%)	0,063	1 (0,40%)	3 (1,19%)	37,75	8 (3,16%)	7 (2,77%)	8 (3,16%)	15 (5,93%)
**Posição**									
Atacante	86 (33,99%)	0,680	23 (9,09%)	5 (1,98%)	10,92	23 (9,09%)	32 (12,65%)	30 (11,86%)	3 (1,19%)
Meia	44 (17,39%)	0,348	17 (6,72%)	4 (1,58%)	15,86	1 (0,40%)	16 (6,32%)	23 (9,09%)	4 (1,58%)
Volante	33 (13,04%)	0,261	8 (3,16%)	2 (0,79%)	13,52	0	7 (2,77%)	25 (9,88%)	1 (0,40%)
Lateral	50 (19,76%)	0,396	17 (6,72%)	4 (1,58%)	14,32	4 (1,58%)	4 (1,58%)	25 (9,88%)	17 (6,72%)
Zagueiro	30 (11,85%)	0,237	9 (3,56%)	3 (1,19%)	22,50	1 (0,40%)	10 (3,95%)	15 (5,93%)	4 (1,58%)
Goleiro	10 (3,95%)	0,079	5 (1,98%)	0 (0%)	5,6	0	1 (0,40%)	3 (1,19%)	6 (2,37%)

Obs: As porcentagens estão descritas em relação ao total de lesões do tornozelo avaliadas. A média de idade está escrita em anos. A média de afastamento está escrita em dias.

A média de idade dos jogadores lesionados foi de 25,94 anos, sendo que as diferentes lesões apresentaram distribuição semelhante entre as faixas etárias. Exames de imagem foram solicitados em 94,07% das lesões, totalizando 391 exames realizados ( Tabela 2 ).

**Tabela 2:** Exames de imagem solicitados para cada diagnóstico de lesão

Diagnóstico	Nenhum	RNM	Radiografia	RX + RNM	RX + TC + RNM	Total geral
Entorse simples	15	9	48	1	0	73
Fratura tornozelo bimaleolar	0	0	2	0	0	2
Fratura tornozelo unimaleolar	0	0	8	0	0	8
Lesão ligamentar lateral	0	8	0	127	1	136
Lesão ligamentar medial	0	4	0	22	0	26
Lesão da sindesmose	0	3	4	1	0	8
Total geral	15	24	62	151	1	253

Abreviaturas: RNM, ressonância nuclear magnética; RX, radiografia; TC, tomografia computadorizada.

## Posição do Jogador, Local no Campo e Momento da Lesão

A posição com maior número de lesões foi a de atacante, seguida pelos laterais. As posições com menor número de lesões de tornozelo foram os goleiros e zagueiros ( Tabela 1 ). Em relação ao total de jogadores da mesma posição lesionados, as entorses do tornozelo apresentaram maior prevalência em volantes ( Tabela 3 ).

**Tabela 3:** Relação da posição dos jogadores com lesões de tornozelo

Diagnóstico	Posição					
	Atacante	Meia	Volante	Lateral	Zagueiro	Goleiro
Entorse simples	25 (9,88%)	12 (4,74%)	6 (2,37%)	16 (6,32%)	8 (3,16%)	6 (2,37%)
Fratura tornozelo bimaleolar	0	1 (0,40%)	6 (5,61%)	0	1 (0,40%)	0
Fratura tornozelo unimaleolar	2 (0,79%)	1 (0,40%)	0	3 (1,19%)	1 (0,40%)	0
Lesão ligamentar lateral	46 (18,18%)	21 (8,30%)	0	26 (10,28%)	16 (6,32%)	3 (1,19%)
Lesão ligamentar medial	11 (4,35%)	3 (1,19%)	0	3 (1,19%)	4 (1,58%)	1 (0,40%)
Lesão da sindesmose	2 (0,79%)	1 (0,40%)	5 (1,98%)	2 (0,79%)	0	0

O meio de campo foi o local com maior número de entorses (75,50%). Apenas 13,04% das lesões ocorreram na área de defesa e 11,46% na área de ataque ( Tabela 1 ). A posição do jogador foi estatisticamente associada com o local do campo de ocorrência da lesão ( *p* < 0,001), sendo que o meio de campo defensivo foi o local mais comum de entorse para todas as posições, exceto para atacantes (mais entorses do meio de campo ofensivo) e goleiros (área defensiva). O final de cada tempo regulamentar foi responsável pelo maior número de lesões, sendo que 47,04% das lesões ocorreram entre 31 e 45 min e 75 e 90 min ( Tabela 4 ).

**Tabela 4:** Relação do tempo de jogo em que ocorreram as lesões com a posição dos jogadores

Posição	Tempo de jogo							
	0–15min	16–30min	31–45min	1° Acréscimo	46–60min	61–75min	76–90min	2° Acréscimo
Atacante	4 (1,58%)	14 (5,53%)	20 (7,91%)	10 (3,95%)	4 (1,58%)	10 (3,95%)	22 (8,70%)	2 (0,79%)
Meia	6 (2,37%)	8 (3,16%)	9 (3,56%)	1 (0,40%)	3 (1,19%)	6 (2,37%)	9 (3,56%)	0
Volante	2 (0,79%)	5 (1,98%)	6 (2,37%)	0	6 (2,37%)	1 (0,40%)	12 (4,74%)	0
Lateral	5 (1,98%)	6 (2,37%)	16 (6,32%)	5 (1,98%)	4 (1,58%)	4 (1,58%)	9 (3,56%)	1 (0,40%)
Zagueiro	4 (1,58%)	2 (0,79%)	5 (1,98%)	3 (1,19%)	3 (1,19%)	8 (3,16%)	5 (1,98%)	0
Goleiro	1 (0,40%)	1 (0,40%)	1 (0,40%)	1 (0,40%)	1 (0,40%)	0	5 (1,98%)	0
Total	22 (8,70%)	36 (14,23%)	57 (22,53%)	20 (7,91%)	21 (8,30%)	25 (9,88%	62 (24,51%)	3 (1,19%)

## Reincidência, Cirurgia e Afastamento

As lesões foram reincidentes em 31,22% dos casos, sendo as entorses simples (38,36%) e a LLL (32,35%) as duas que apresentaram maiores taxas de reincidência, enquanto as fraturas não tiveram reincidência ( Tabela 1 ).

As lesões foram tratadas de forma cirúrgica em 7,11% dos casos, com destaque para fratura do tornozelo unimaleolar, que representou 33,33% do total de cirurgias. Nenhuma entorse simples de tornozelo foi tratada cirurgicamente e apenas 3,68% das LLLs e 7,69% das LLMs foram tratadas cirurgicamente. As lesões reincidentes foram tratadas cirurgicamente em 12,65% dos casos, enquanto as não reincidentes em 80% dos casos ( Tabela 1 ).

As lesões foram classificadas de acordo com a gravidade conforme o tempo de afastamento do atleta, da seguinte forma: lesão leve (< 3 dias), menor (3–7 dias), moderada (8–28 dias), maior (4–8 semanas), grave (> 8 semanas). O tempo médio de afastamento foi de 13,95 dias, sendo que as lesões graves e maiores foram responsáveis por 4,34% e 3,56% do total de lesões, respectivamente ( Tabela 5 ).

**Tabela 5:** Gravidade das lesões de tornozelo

Diagnóstico	Gravidade				
	Leve	Menor	Moderada	Maior	Grave
Entorse simples	38 (15,02%)	15 (5,93%)	20 (7,91%)	0	0
Fratura tornozelo bimaleolar	0	0	0	0	2 (0,79%)
Fratura tornozelo unimaleolar	0	1 (0,40%)	0	1 (0,40%)	6 (2,37%)
Lesão ligamentar lateral	32 (12,65%)	44 (17,39%)	55 (21,74%)	4 (1,58)	1 (0,40%)
Lesão ligamentar medial	4 (1,58%)	10 (3,95%)	0	1 (0,40%)	1 (0,40%)
Lesão da sindesmose	0	0	3 (1,19%)	4 (1,58%)	1 (0,40%)
Total	74 (29,25%)	70 (27,67%)	78 (30,83%)	10 (3,95%)	11 (4,35%)

## Discussão

Os principais achados do presente estudo foram a alta incidência de entorses de tornozelo ao longo dos 4 anos. Além disso, as entorses acometeram principalmente atacantes e volantes, com maior ocorrência no meio de campo e nos 15 minutos finais de cada tempo regulamentar. A taxa de reincidência foi elevada (31,22%) e o tempo médio de afastamento foi cerca de 2 semanas.

Ekstrand et al. 4 afirmaram que as entorses do tornozelo foram responsáveis por 17 a 21% de todas as lesões envolvendo a prática esportiva, o que mostra sua grande representatividade. Em nosso estudo identificamos um índice FIFA de 2,00 para as entorses de tornozelo, um valor superior às lesões do membro superior (índice FIFA = 1,34) 5 e rupturas do ligamento cruzado anterior (índice FIFA = 0,41), 6 porém inferior às lesões musculares (índice FIFA = 7,66). 7

Em um estudo alemão que avaliou atletas da Bundesliga (liga alemã de futebol profissional) nas temporadas de 2008 a 2014, foram observados 280 episódios de entorses do tornozelo (5,17% do total de lesões) com uma taxa de 46,66 lesões por ano, o que representa uma diferença de 35,55% em relação ao nosso estudo. 3 Árnason et al. 8 avaliaram 10 clubes da elite do futebol islandês, e do total de lesões encontradas, 82% localizavam-se nos membros inferiores.

Dauty et al. 9 não encontraram diferença na incidência de lesões durante as 15 temporadas analisadas entre 1995 e 2010, o que contrasta com os resultados apresentados no atual estudo. Não há dados que informem o motivo da diminuição do quadro de lesões em nosso estudo; porém, pode-se observar que, ao longo dos anos, a qualidade da preparação física pré-jogo, fisioterapia, qualidade do gramado e tratamentos de prevenção de lesões teve melhora significativa no futebol brasileiro, o que pode ter colaborado para a diminuição das lesões. 2 10

Avaliando as distribuições das lesões por mês, Kofotolis et al. 11 observaram que 47,48% das entorses do tornozelo aconteceram no mês de agosto e setembro. Morgan e Oberlander 12 observaram que 24% ocorreram no primeiro terço da temporada, 25% no segundo terço da temporada e 29% no último terço da temporada, o que também entra em confronto com os dados do presente estudo.

No estudo de Nery et al. 13 as lesões ligamentares (medial e lateral) do tornozelo representaram 66,8% do total de lesões sofridas do tornozelo durante as 2 temporadas avaliadas. Comparando com o estudo atual, 63,93% de todas as lesões do tornozelo foram por lesões do complexo medial e lateral, o que contribui para a afirmação de que lesões do complexo medial e lateral são comuns nos casos de entorse do tornozelo.

Árnason et al. 8 observaram que 29% das contusões ocorreram por lesões musculares, 22% por lesões ligamentares e 20% por traumas contusos diretos. E ao avaliar as lesões do tornozelo, a maioria evoluiu com lesões no complexo lateral do tornozelo.

Em uma revisão sistemática, de todas as lesões do tornozelo no futebol, 76,8% foram causadas por entorses, que é a causa mais comum de lesões do tornozelo, o que corrobora o atual estudo. 4 Nery et al. 13 indicaram que o mecanismo de trauma mais comum no atleta profissional de futebol foi o trauma direto entre jogadores (32%), sendo que o uso excessivo (mobilidade articular intensa e no extremo do arco de movimento) foi associado a 26% das causas das lesões do tornozelo. Kofotolis et al. 11 também afirmaram que a maioria das lesões do tornozelo ocorreram por contato direto com outro atleta, com uma taxa de 63,3%, enquanto 36,7% foram lesões sem contato. Na Copa do Mundo de 2014, 64,4% das lesões dos atletas foram por contato. 14

A posição do atleta em campo mais associada à entorse do tornozelo foi a de atacante. Porém, se considerarmos as posições meia e volante como meio-campistas em geral, observa-se uma maior taxa de entorse simples nessa categoria (7,11%). Cohen et al. 2 compararam as lesões totais do corpo dos atletas, e observaram que a posição de meio-campo teve maior número de lesões, assim como Leventer et al. 15 e Morgan e Oberlander, 12 que mostraram que na *Major League of Soccer* (MLS), os meio-campistas tiveram 37,6% de ocorrências de lesões totais pelo corpo.

Dauty et al. 9 não indicaram nenhuma diferença da quantidade de quaisquer tipos de lesões em diferentes tipos de posições; porém, houve uma leve tendência para zagueiros. Já Kofotolis et al. obtiveram mais lesões em jogadores da defesa (zagueiros e laterais) (42,4%) em comparação com os meio-campistas (32,3%) e atacantes (20,8%). 11

Kofotolis et al. 11 analisaram que 61,1% das lesões do tornozelo ocorreram nos 15 minutos finais tanto no primeiro tempo quanto no segundo tempo do jogo, concordando com os dados do presente estudo. Esse mesmo padrão também foi encontrado por Hawkins et al. 16

No presente estudo, 31,22% das lesões foram reincidentes. Árnason et al. 8 observaram que 35% das lesões foram reincidentes; desses, 8% ocorreram em menos de 1 mês após a lesão anterior. Kofotolis et al. observaram que 60,5% dos atletas tiveram lesões reincidentes. 11 Jogadores de futebol com histórico de entorses do tornozelo prévias tem 4,9 vezes mais chances de ter uma reincidência. 13

Cohen et al. 2 avaliaram episódios de entorse, sendo que 5,2% (n = 9) dos casos evoluíram para algum procedimento cirúrgico, de um total de 173 casos de entorse. Segundo Nery et al., 13 20% dos atletas com entorse do tornozelo agudas vão evoluir com instabilidade mecânica ou funcional do tornozelo, resultando em uma instabilidade crônica do tornozelo, com indicação de tratamento cirúrgico.

Kofotolis et al. 11 também avaliaram o tempo de afastamento. Observou-se que as entorses simples representaram a lesão mais comum com menos de 7 sessões perdidas. A lesão do ligamento fibulotalar anterior teve a maior taxa de afastamento como causa, com 58,7% do tempo total perdido por lesão.

Morgan e Oberlander 12 separaram em três categorias a severidade da lesão. Das 256 lesões encontradas, 60% foram classificadas como leves, 26% como moderadas e 14% como graves. Árnason et al. 8 classificaram as lesões em quatro categorias de acordo com o tempo de afastamento, sendo que a maioria dos atletas retornaram ao esporte em menos de 2 semanas. Cohen et al. 2 observaram que 49,7% das entorses obtiveram afastamento de menos de 7 dias, 45% entre 7 e 30 dias e 5,2% em mais de 30 dias, sendo estes últimos todos casos cirúrgicos.

A principal limitação deste estudo é o possível viés de coleta de dados, inerente a estudos epidemiológicos, uma vez que os médicos dos clubes foram os responsáveis pelo preenchimento dos dados na plataforma. Nesse período houve algumas trocas de médicos nos clubes e para reduzir o viés de coleta de dados, todos os médicos foram treinados para preencher a pesquisa. Sempre que o médico do clube era substituído, nós fomos informados e o novo médico também foi treinado. Outra limitação é que não há consenso sobre a definição de lesão na literatura, dificultando a comparação de nossos resultados com os de outros estudos. Além disso, não foram coletados dados sobre lesões ocorridas durante as sessões de treinamento. Por fim, a não avaliação da qualidade dos gramados e sua relação com as entorses também foi uma limitação do estudo. Apesar das limitações, trata-se de um extenso estudo prospectivo com duração de 4 anos e grande amostra de atletas e lesões, no qual foi possível coletar dados inéditos de dois grandes campeonatos de futebol profissional no Brasil.

## Conclusão

As entorses de tornozelo são lesões frequentes no futebol. Apesar do tempo médio para retorno ao esporte ser breve, essas lesões apresentam grande taxa de reincidência e são potencialmente cirúrgicas, o que está associado a maior tempo de afastamento. As lesões mais comuns foram as lesões ligamentares laterais e as entorses simples. As posições mais acometidas foram os atacantes e volantes, com maior ocorrência no meio de campo e nos 15 minutos finais de cada tempo regulamentar.
